# Stress mitigation by riparian flora in industrial contaminated area of River Chenab Punjab, Pakistan

**DOI:** 10.7717/peerj.15565

**Published:** 2023-06-28

**Authors:** Toqeer Abbas, Iftikhar Ahmad, Zafar Iqbal Khan, Anis Ali Shah, Ryan Casini, Hosam O. Elansary

**Affiliations:** 1 Department of Botany, University of Sargodha, Sargodha, Punjab, Pakistan; 2 Department of Botany, Division of Science and Technology, University of Education, Lahore, Punjab, Pakistan; 3 School of Public Health, University of California, Berkeley, CA, USA; 4 Department of Plant Production, College of Food & Agriculture Sciences, King Saud University, Riyadh, Saudi Arabia

**Keywords:** Riparian flora, Industrial pollution, River Chenab, Effluents, Heavy metals, Water, Soil, Phytoremediation

## Abstract

Faisalabad is a major industrial area in Pakistan’s Punjab province that discharges wastewater into the Chenab River. Industrial effluents in Faisalabad are predicted to pose a significant threat to the riparian vegetation of the Chenab River and nearby vegetation. Heavy metal pollution of plants, water, and soils is one of the biggest problems worldwide that needs to be addressed because heavy metals above normal levels are extremely dangerous to both riparian vegetation and wildlife. The results indicated high levels of pollution in the industrial effluents as well as in the river in terms of salinity, metal toxicity, TSS, TDS, SAR, the acidic and alkaline nature of the industrial effluents, and the spread of industrial effluents up to 15 square kilometres in the Chenab River. Despite the higher pollution, four plants were found at all sites: *Calotropis procera*, *Phyla nodiflora*, *Eclipta alba* and *Ranunculus sceleratus*. It was found that most of the selected plants were phytoaccumulators, making them best suited to survive in harsh environments such as those with industrial pollution. The Fe concentration in the plant constituents was the highest, along with Zn, Pb, Cd, and Cu, all of which were above the permissible limits of the WHO. The metal transfer factor (MTF) was higher in most of the plants studied, and even exceeded 10 at some severely affected sites. *Calotropis procera* proved to be the most suitable plant for growth on drainage systems and also at river sites, as it had the highest importance value across all sites and seasons.

## Introduction

Riparian zones serve as a barrier between the aquatic and terrestrial environments. Riparian zones provide many ecosystem services, such as nutrient modification, erosion control and temperature regulation, which improve water quality in adjacent aquatic ecosystems (Saklaurs et al., 2022).

The textile industry is a major source of environmental pollution. In terms of environmental pollution, the textile processing industry tops the list. Although the industrial area of Faisalabad is the backbone of Pakistan’s economy, these industries consume a large amount of water and generate excessive wastewater. This wastewater is discharged without treatment into water canals, where it flows into the Chenab River (Hossain et al., 2010).

Heavy metals are the most prevalent pollutants in industrial effluents. One method of cleaning heavy metal contaminated soils is to use plants that have the ability to grow, adapt and absorb metals (Budovich, 2021). The pH of the water and the solubility of the metals increased and the metal particles became more mobile. This is the reason why metals are more toxic in soft waters (Zhang, Zeng & Cheng, 2016). Heavy metals such as cadmium (Cd), chromium (Cr), copper (Cu), iron (Fe), manganese (Mn), nickel (Ni), potassium (K), phosphorus (P), sodium (Na), sulphur (S) and zinc (Zn) were present at concentrations above the recommended NEQS. As a result, it was determined that textile effluents were significantly contaminated (Imtiazuddin, Mumtaz & Mallick, 2012).

Studies showed that the accumulation of Cr was much higher in *Calotropis procera* compared to other plants, with concentrations as high as 188.2 and 68.2 mg/kg in shoots and roots, respectively (Usman et al., 2020). Cadmium concentrations of 13.4% and 22.3% were found in the leaves of *C. procera* in a study conducted along Faisalabad and Sargodha roads, respectively (Khalid et al., 2018). The ability of *R. sceleratus* to accumulate Mn, Cu, Ni and Pb in its roots indicates the potential use of this plant species for phytostabilization of these heavy metals in polluted water bodies such as rivers and streams (Farahat & Galal, 2018). A relatively short life cycle combined with a high biomass accumulation rate makes *R. sceleratus* useful for dynamically constructed wetlands aimed at treating concentrated wastewater, according to a study on its phytoextraction potential (Ievinsh et al., 2022).

This study presents phytoremediation as one of the bioremediation methods for soils contaminated with organic and inorganic compounds. The results suggest that in order to reduce soil contamination and the resulting poisoning of food and humans, it is better to identify the factors that lead to increased exposure to heavy elements in the environment of the given area and then select plants that are not consumed as food before planting crops.

## Materials and Methods

### Study area

The Chenab River flows about 46 km from Faisalabad in Pakistan’s Punjab province through a mosaic of scattered plains, agriculture, urban areas and mountains. These plains and mountains, which originated in Jammu and Kashmir, cover an area of about 960 km^2^. Faisalabad, a city with textile and dyeing mills and industries that print, weave, dye and finish fabrics, is one of the most productive cities in Pakistan (Nosheen, Nawaz & Rehman, 2000).

### Riparian sites selection

Ten sites (nine polluted and one unpolluted) were selected to study water and soil parameters and metals in water, soil and plants of some dominant species. The polluted sites were selected within a radius of 500 m from the polluted area, while the unpolluted area was about 14 km away near the Chenab Nagar hills. While selecting the sites for plant collection, care was taken to minimize disturbance (by humans and animals) to ensure the consistent accumulation of industrial pollutants at the plant sites.

### Study area climate

The study area consists of dry plains and a foothill, where the climate is dry and hot. The average maximum temperature in June was 48 °C and the minimum temperature in January was 4 °C (Pakistan Metrological Department, accessed March 2019). The climatic conditions in the study area fall under the warm desert climate (BWh) according to the Koeppen-Geiger classification. The average annual rainfall is 336 mm in Chiniot and 346 mm in Faisalabad.

### Collection of samples and statistical analysis

The roots, stems and leaves of selected plants (*Calotropis procera*, *Eclipta alba*, *Phyla nodiflora* and *Ranunculus sceleratus*) were collected from the selected sites and carefully brought to the laboratory for metal analysis. Water and soil samples were also taken at the 10 selected sites in four seasons.

The experiment was conducted in a 2-factorial (collection areas and species) completely randomized design with six replicates. The means were compared using Duncun’s Multiple Range Test at a significance level of *p* ≤ 0.05. Principal component analysis (PCA) was performed and biplots were generated using Cano Draw *v.* 4.0 supplied with CANOCO for Windows version. 4.5.

## Results

### Industrial effluents and riverine water analysis

Water samples were collected from all three drains and seven different sites (one non-polluted) of the Chenab River during four seasons. The metal values are determined to check the heavy metal toxicity in the water samples. The samples were digested for heavy metal determination. The concentration of various metals in industrially polluted drains showed significant differences between the different polluted sites. The polluted sites received much higher effluents compared to the unpolluted control.

The analysis of the physico-chemical properties of the effluents from ten sites revealed that the pH was highest in the Paharang drain. The data show that the pH was above 7 at studied sites. The electrical conductivity of the effluents differed either among the three drains or among the seven sites. The highest value of EC was recorded at the Paharang drain with a value of 48.27, followed by the Marah Chiniot drain and Ahmad wala drain with values of 47.38 and 45.24, respectively. The values of chemical oxygen demand (COD) and biological oxygen demand (BOD) differed greatly among the pollution levels and were high in three drains compared to the non-polluted and river sites (Table 1).

**Table 1:** Comparison of means of physiochemical properties of water parameters from 10 different studied sites.

Site	pH	EC (mS/cm)	COD (mg/l)	BOD (mg/l)
Non-polluted	7.13 ± 0.06e	19.00 ± 1.19d	130.25 ± 7.76g	159.00 ± 2.10f
Marah drain	7.78 ± 0.06c	47.38 ± 1.00a	312.25 ± 7.50ab	236.75 ± 7.56c
River site 2	8.25 ± 0.03b	24.77 ± 1.87c	276.25 ± 12.57c	166.50 ± 7.81ef
River site 3	8.28 ± 0.09b	32.43 ± 1.00b	242.50 ± 5.15d	177.25 ± 8.81e
Paharang drain	8.65 ± 0.03a	48.27 ± 3.03a	301.25 ± 7.40b	311.75 ± 5.82a
River site 4	7.15 ± 0.03e	33.53 ± 1.12b	212.00 ± 8.35e	217.00 ± 2.10d
River site 5	7.13 ± 0.04e	24.90 ± 1.22c	180.00 ± 4.33f	213.75 ± 6.65d
Ah.wala drain	8.20 ± 0.07b	45.24 ± 1.95a	325.25 ± 8.76a	286.50 ± 7.56b
River site 6	7.30 ± 0.05d	30.60 ± 1.07b	213.25 ± 11.77e	183.75 ± 5.22e
River site 7	7.25 ± 0.00de	22.43 ± 0.00cd	168.75 ± 0.00f	180.25 ± 0.00e

Site	Ca+Mg (mEq/L)	Sodium (mEq/L)	Bicorbonatem (Eq/L)	Carbonate (mEq/L)
Non-polluted	5.15 ± 0.06f	33.25 ± 7.16e	6.22 ± 0.85e	5.19 ± 0.43f
Marah drain	9.75 ± 0.10bc	391.75 ± 0.25c	13.75 ± 0.37d	15.99 ± 0.09b
River site 2	8.68 ± 0.07e	38.25 ± 0.48e	7.08 ± 0.23e	9.73 ± 0.15c
River site 3	8.53 ± 0.45e	36.75 ± 15.27e	6.98 ± 1.06e	8.60 ± 0.71d
Paharang drain	11.13 ± 0.16a	531.75 ± 7.59b	21.03 ± 0.82a	17.27 ± 0.01a
River site 4	9.42 ± 0.12cd	37.25 ± 3.77e	17.84 ± 0.51b	9.09 ± 0.12cd
River site 5	8.73 ± 0.44e	34.25 ± 84.44e	15.58 ± 0.72c	8.60 ± 0.55d
Ah.wala drain	10.34 ± 0.19b	1,455.00 ± 0.63a	19.80 ± 0.26a	17.12 ± 0.17a
River site 6	8.65 ± 0.10e	152.25 ± 1.03d	18.14 ± 0.39b	4.88 ± 0.11f
River site 7	9.00 ± 0.00de	151.75 ± 0.00d	18.10 ± 0.00b	6.91 ± 0.00e

Site	Chlorides (mEq/L)	SAR	TDS (mS/cm)
Non-polluted	12.39 ± 0.39g	20.85 ± 3.12e	819.3 ± 66.7cd
Marah drain	33.78 ± 0.32a	177.66 ± 0.09c	1,418.5 ± 12.3b
River site 2	23.99 ± 0.29de	18.35 ± 0.28e	859.3 ± 29.4cd
River site 3	22.14 ± 0.71f	17.91 ± 2.50e	845.8 ± 129.6cd
Paharang drain	34.64 ± 0.41a	225.94 ± 3.39b	1,358.3 ± 24.8b
River site 4	29.10 ± 0.34b	17.10 ± 1.80e	827.3 ± 12.0cd
River site 5	26.75 ± 0.81c	16.46 ± 40.28e	965.3 ± 55.0c
Ah.wala drain	34.37 ± 0.26a	642.54 ± 0.77a	1,679.5 ± 50.4a
River site 6	25.04 ± 0.42d	73.39 ± 0.30d	807.8 ± 17.7d
River site 7	23.17 ± 0.00ef	72.26 ± 0.00d	837.5 ± 0.0cd

**Notes.**

Means sharing similar letter in a column are statistically non-significant (*P* > 0.05).

Ca+Mg concentrations were high at all drain sites in this study compared to river sites. The highest value of Ca+Mg ions was measured in the water samples from the Paharang drain. Sodium levels were excessive at all drains compared to river sites, with the highest enrichment found at the Paharang drain. Bicarbonate ions in the textile dye industry effluents were highest in the Paharang drain, as the Paharang drain contains most of the effluents from the industrial units. As a result of the dilution of industrial water by the Chenab River, lower bicarbonate content was measured at River Site 1 and at River Site 3. The amount of carbonate in the water samples was highest in the Paharang drain. The Parahang drain and Ahmad wala drain were rich in chlorides and carbonates, with maximum values of 34.64 and 34.37 respectively. The SAR and TDS maximum value was recorded in the Ahmad wala drain (Table 1).

**Figure 1:**
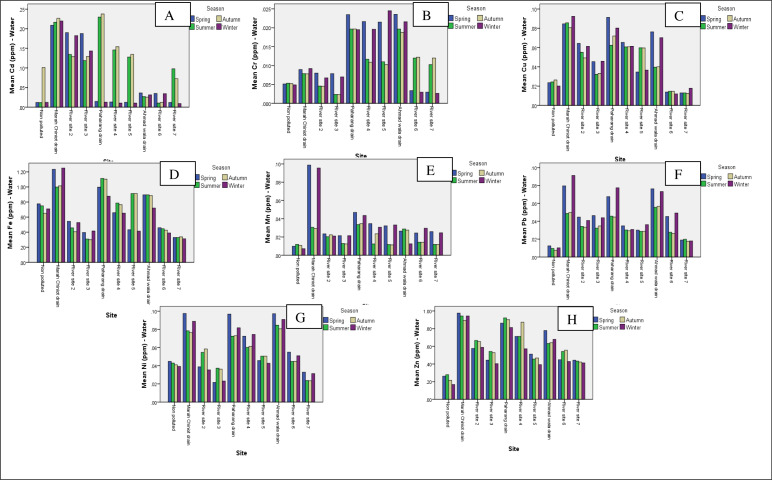
Graphs showing the means for analysis of metals for water from 10 sites. Graphs showing the means for analysis of metals for Cd (A)), Cr (B), Cu (C), Fe (D), Mn (E), Pb (F), Ni (G) and Zn (H) for water from 10 different sites.

The maximum Cd content was found at the Marah Chiniot drain at 0.218 ppm. The safe limits for Cd in effluents recommended by WHO are 0.003 ppm (Aneyo et al., 2016), while decreased Cd level was recorded in the collected samples at River Site 6 at 0.023 ppm, thus the investigation in this study indicates that the Cd content was above the critical level (Fig. 1A). Cr accumulation was high in water samples from Paharang drain, but the lowest value was recorded at River Site 1, a non-polluted site (Fig. 1B). The effluent from the local dye industry showed the highest Cu content in the discharge of the Marah Chiniot drain at 0.086 ppm followed by Ahmad wala drain (Fig. 1C). The Fe concentrations in the drains carrying industrial effluents were particularly high in the Marah Chiniot drain at 1.124 ppm, followed by the Paharang drain at 1.022 ppm. The decreased Fe concentration was observed at site 10 at 0.327 ppm (Fig. 1D).

The highest Mn concentration was found in the Marah Chiniot drain at 0.064 ppm (Fig. 1E). On the other hand, the highest accumulation of Pb was recorded in the Marah Chiniot drain and the decreased Pb load was recorded at non-polluted (Fig. 1F). Moreover, the Ni concentration at three drains was high compared to the river sites, as the flow of industrial effluents was greater at drains (Fig. 1G). The highest level of Zn contamination was found in the Marah Chiniot drain, followed by the Paharang drain. The lowest zinc concentration was quantified at non-polluted site (on the other hand, the highest accumulation of Pb was recorded in the Marah Chiniot drain and the lowest Pb load was recorded at non-polluted (Fig. 1H). The polluted sites had significantly higher concentrations of Cd, Cr, Mn, Pb and Zn in their water compared to the control site. The results depicted that the concentrations of the metals vary depending on the season and location, as previously reported (Zubair et al., 2021; Angaru et al., 2021; Ajiboye, Oyewo & Onwudiwe, 2021) in studies on industrial effluents from different areas of the world.

### Comparison of means among three areas (non-polluted, three drains, and river sites) and four seasons for analysis of metals

**Figure 2:**
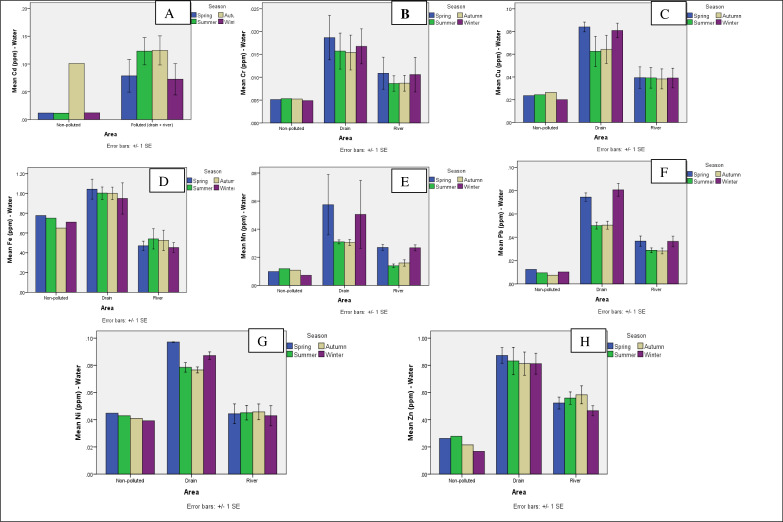
Graphs showing the means for analysis of metals for water. Graphs showing the means for analysis of metals for comparison of a non-polluted, drains and six river sites for Cd (A), Cr (B), Cu (C), Fe (D), Mn (E), Pb (F), Ni (G) and Zn (H) for water.

The graphical data for the analysis of heavy metals from industrial effluents showed that the correlation factor between non-polluted and polluted sites was very high for documented metals except iron. The data show that heavy metals such as Cd, Cr, Cu, Fe, Mn, Ni, Pb and Zn were present at all polluted sites in four seasons with the highest correlation, while the concentration of the metals in the same seasons was lower at non-polluted site. Studies showed that Cd concentrations were higher at polluted sites compared to non-polluted site in all seasons (Fig. 2A). Meanwhile, Cr concentration was higher in four season at all polluted sites mostly at drains, while it was lower in all seasons and at non-polluted site (Fig. 2B). The amount of Cu was higher than 0.04 ppm in all seasons at drains (Fig. 2C). The Fe was also measured in higher concentrations in four seasons at three studied drains (Fig. 2D). The Mn concentration was also elevated at polluted sites compared to non-polluted site in four seasons (Fig. 2E). Moreover, Pb was also present in the highest concentration at polluted sites compared to non-polluted site in four seasons (Fig. 2F). The Ni showed similar variations as those of Cd, Cr, Cu, and Mn in the graphic data and was reported in higher content at polluted sites than at non-polluted site (Fig. 2G). The potential of Zn showed the same variation as shown by other metals (Fig. 2H). Arsenic, cadmium, chromium, copper, lead, mercury, nickel, and zinc are dangerous, carcinogenic metals that accumulate in living organisms. Water polluted with HMs causes additional major health issues; even trace concentrations of HMs in water can have negative environmental implications (Singh et al., 2022).

### Soil analysis

Data collected from the industrially influenced drains and river sites revealed that phosphorus levels were highest in the Paharang drain with value of 27.80, followed by the Ahmad wala drain and the least enrichment was recorded at River Site 4 with a concentration of 6.21. The amount of P was also found to be high in the Marah Chiniot drain. The analysis of soil samples from the area revealed that the amount of potassium was the highest in the Paharang drain with a value of 241.50, followed by the Ahmad wala drain. The saturation of the soil samples was highest at four different sites with no such changes, with a value of 42.0 at River Site 2, River Site 4, River Site 6 and River Site 7. The lowest saturation value was recorded in River Site 1 and River Site 3 (Table 2).

**Table 2:** Comparison of means of physiochemical properties of soil parameters.

Site	pH	EC (mS/cm)	Organic matter
Non-polluted	7.10 ± 0.02 g	13.25 ± 0.18ab	1.46 ± 0.03bc
Marah drain	7.78 ± 0.03d	10.70 ± 0.12d	2.16 ± 0.01a
River site 2	8.28 ± 0.06a	12.79 ± 0.90b	1.37 ± 0.16bcd
River site 3	8.28 ± 0.03a	10.96 ± 0.19d	1.64 ± 0.01b
Paharang drain	7.88 ± 0.00c	9.38 ± 0.06e	2.14 ± 0.16a
River site 4	7.40 ± 0.00e	11.18 ± 0.05cd	1.42 ± 0.02bcd
River site 5	7.20 ± 0.04f	12.46 ± 0.41b	1.16 ± 0.00d
Ah.wala drain	8.10 ± 0.00b	12.46 ± 0.20b	2.19 ± 0.05a
River site 6	7.20 ± 0.00f	14.36 ± 0.03a	1.20 ± 0.13cd
River site 7	7.10 ± 0.00 g	12.36 ± 0.00bc	1.32 ± 0.00cd

Site	P mg/kg	K mg/kg	Saturation
Non-polluted	7.99 ± 0.44e	112.25 ± 4.14f	40.50 ± 0.25c
Marah drain	21.20 ± 0.24b	231.00 ± 1.80a	41.75 ± 0.00ab
River site 2	9.41 ± 0.19d	191.25 ± 11.35c	42.00 ± 0.50a
River site 3	10.17 ± 0.56d	155.75 ± 10.94d	40.50 ± 0.71c
Paharang drain	27.80 ± 0.20a	241.25 ± 2.72a	41.00 ± 0.00bc
River site 4	6.21 ± 0.27f	209.50 ± 5.71b	42.00 ± 0.25a
River site 5	9.54 ± 0.58d	184.25 ± 4.38c	41.75 ± 0.25ab
Ah.wala drain	27.18 ± 0.25a	232.00 ± 4.02a	41.75 ± 0.00ab
River site 6	11.61 ± 0.18c	142.00 ± 2.95de	42.00 ± 0.00a
River site 7	11.65 ± 0.00c	125.75 ± 0.00ef	42.00 ± 0.00a

**Notes.**

Means sharing similar letter in a column are statistically non-significant (*P* > 0.05).

The soil potassium content was higher than the standard values (80 mg/kg) for K in the soil established by Rhue & Kidder (1983). The role of K in the soil is noteworthy. K plays a role as a regulator of plant growth function. It is an essential component of protein and carbohydrate synthesis. The analysis of soil samples from ten different sites in four different seasons revealed that industrial effluents transported through the sewerage system in the form of contaminated water severely affect the area along the confluence with the Chenab River. It was also found that three drains, namely the Marah Chiniot drain, the Paharang drain and the Ahmad wala drain, which flow into the Chenab River, have increased the affected area of the river.

**Figure 3:**
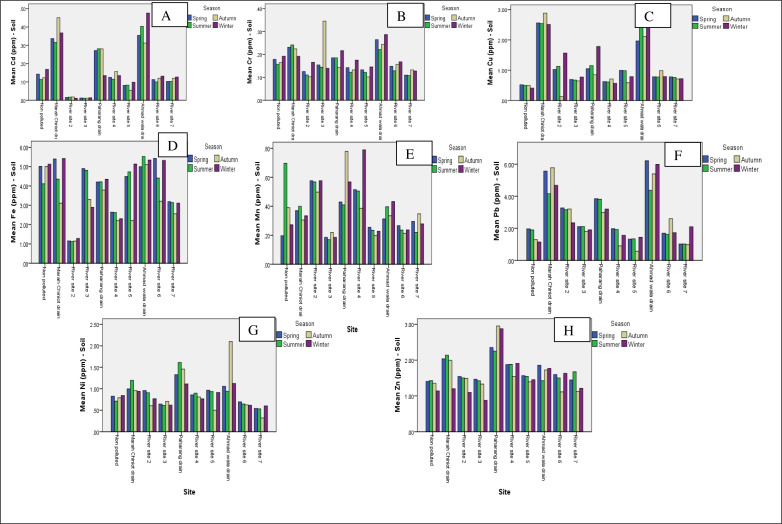
Graphs showing the means for analysis of Cd (A), Cr (C), Cu (C), Fe (D), Mn (E), Pb (F). Ni (G) and Zn (H) for soil metals from 10 sites.

According to soil samples taken from various locations, the Ahmad wala drain has the highest levels of cadmium and chromium contamination. The Marah Chiniot drain exhibited considerable levels of Cd with 0.368 ppm and Cr 0.223 ppm respectively (Figs. 3A & 3B). According to WHO (1998), this study found a higher Cr level than the standard. The contamination graph for Cu was greater in the Marah Chiniot and Ahmad wala drain (Fig. 3C). In the present study, the Cu and Cr levels of soil at many sites were above the toxic limit because there were industrial effluent depositions in selected areas. The soil samples being studied suggest that the maximum Fe contents were measured at Ahmad wala drain (Fig. 3D). Industrial waste and contamination of sediments result in elevated Cd levels in riparian vegetation (Proshad et al., 2022).

The soil parameter results are more reliable for site selection because they include all sites, whether they drain water or not. When compared to other locations, the Ahmad wala drain graph for four seasons indicates a high amount of Pb and Ni (Figs. 3F & 3G). Mn and Zn higher concentration was documented in the Paharang drain (Figs. 3E & 3H). Statistical analyses were conducted separately for each season and site to track changes in physiological and biochemical parameters, as well as heavy metals, at spatial and temporal scales. Graphical data for heavy metals from the soil samples showed variations at each site and in each season, from non-significant to highly significant. The Zn plays a vital role in plants by performing many essential functions like photosynthesis, membrane structure, gene expression and regulation, lipid and nucleic acid metabolism, phytohormone activity, protein synthesis, and defense against disease and drought (Begum et al., 2016). The present investigation showed that the metals concentration in soil samples was similar to that reported earlier by Zhao et al. (2022). Variance of data showed that a highly significant amount of all eight metals under study was present at different sites, while fluctuating very little among the seasons. In China, heavy metal contamination from dye manufacturers is severe, with Pb, Cd, Zn, Ni, and As being the most prevalent contaminants (Peng et al., 2022).

### Comparison of means for soil analysis among three areas (non-polluted, drains and river sites) and four seasons

**Figure 4:**
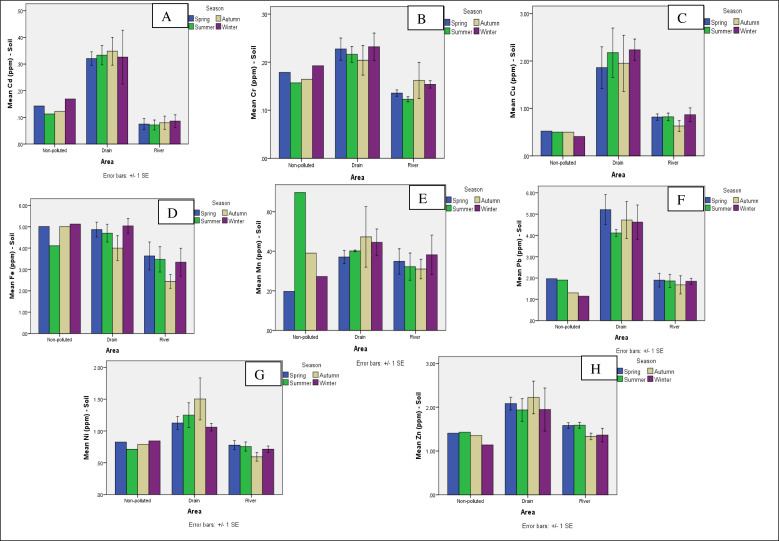
Graphs showing the comparison of means for analysis of Cd (A), Cr (B), Cu (C), Fe (D), Mn (E), Pb (F), Ni (G), and Zn (H) for soil metals from 10 sites.

Graphical data for the comparison of non-polluted site with three drains and six river-polluted sites clearly indicate that the concentration of metals in soil samples was higher in the polluted area compared to the non-polluted area, except for Fe and Mn. Iron showed variations compared to other metals, and iron concentration was higher at non-polluted site in four seasons compared to polluted river sites and drains (Fig. 4D). The metal Cr concentrations were higher in the spring at drains (Fig. 4B). The Mn concentrations were also higher at non-polluted site in summer compared to drains and river sites (Fig. 4E). The graphs for analysis show that the Cd, Cu, Pb, Ni, and Zn concentrations in soil samples were high at drains and the metals were associated with drain sites (Marah Chiniot drain, Paharang drain, and Ahmad wala drain) compared to river sites (Figs. 4A, 4C, 4F, 4G and 4H respectively).

### Water and soil combined biplots

The PCA biplot for the analysis of data for water and soil combined in March to early spring showed Site 2 on extreme with respect to the pH of water, potassium, EC of water, K, COD and saturation. Similarly, Site 5 was clustered close to organic matter, CO3, BOD, Cl, phosphorus and total dissolved solids.

The biplot shows that the combined analysis of soil and water in the summer showed that the biplot data were similar in the summer and spring. CO3, Cl, EC of water, pH of water, Ca+Mg and TDS were present in the highest association with Site 5, while K was found in a highly associated state with Site 2. The biplot for the month of September, which is autumn, shows the same correlation as reported in spring and summer, and Site 2 had the highest correlation with the pH of the water and Ca + Mg ions. Potassium, Cl and CO3 were clustered close to Site 2, while the pH of water, organic matter, EC of water, phosphorus, BOD, and TDS were found to be associated with Site 5. Saturation showed an association with Site 10.

**Figure 5:**
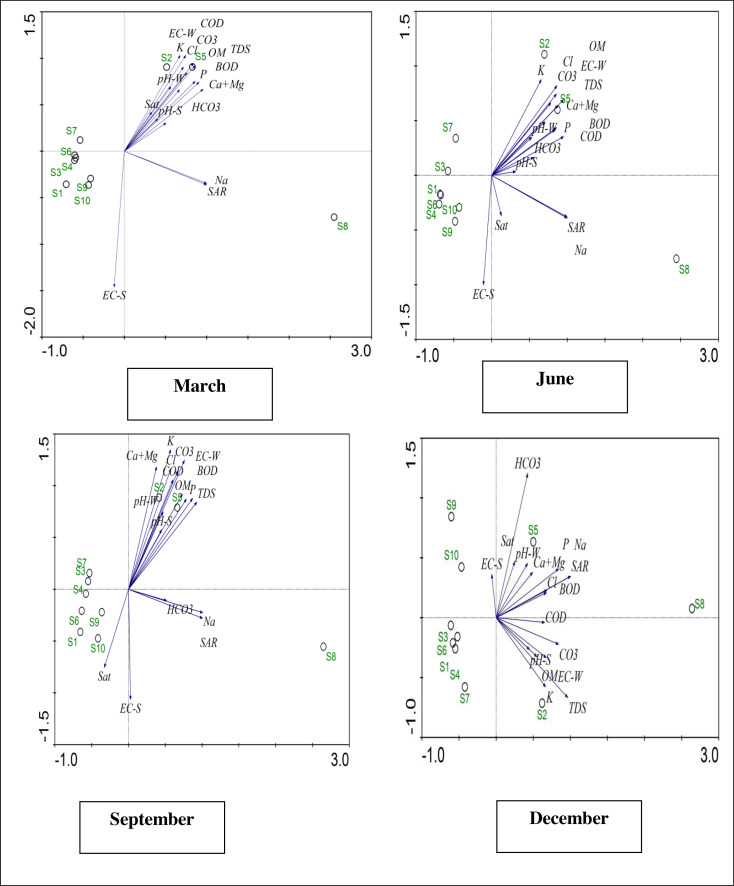
Principal component analysis (PCA) Biplots for water and soil physiochemical analysis in different seasons at 10 sites. Principal Component Analysis (PCA) Biplots for water and soil physiochemical analysis in different seasons at 10 sites. March (spring), June (summer), September (autumn), and December (winter). S1 (River Site 1, non-polluted), S2 (Marah Chiniot drain), S3 (River Site 2), S4 (River Site 3), S5 (Paharang drain), S6 (River Site 4), S7 (River Site 5), S8 (Ahmad wala drain), S9 (River Site 6), S10 (River Site 7).

The biplot for winter was different compared to the biplots for spring, summer and autumn. The data for winter show that EC of soil was associated with Site 10, while pH of water, saturation, Ca+Mg, and phosphorus were associated with Site 5. Site 2 was associated with K, TDS, EC (water) and pH of the soil. The results showed that the parameters of water and soil were on extreme in four seasons at ten different sites (Fig. 5).

### Metals in plant components

In plant components (whole plant) of four ecotypes heavy metals such as Cd, Cr, Cu, Fe, Mn, Ni, Pb and Zn were checked in the three different drains and seven river sites (one non-polluted) throughout the four seasons of the year. The results suggest that the increased cadmium content in all selected plants was measured at the Ahmad wala drain. In plant components, the maximum contamination level of chromium was calculated at River Site 5, followed by the Paharang drain in four ecotypes. Among the plants under study, the highest mean value of Copper was calculated at the Ahmad wala drain. The highest content of iron was accumulated at the Marah Chiniot drain, followed by the Ahmad wala drain. The concentration of Mn was also reported highest in Ahmad wala drain. The maximum nickel value found at the Marah Chiniot drain was 0.665 ppm. The elevated Lead value was calculated at the Marah Chiniot drain at 0.727 ppm in the plants under study. The highest Zinc contamination level at the Ahmad wala drain was calculated to be 1.90 ppm (Table 3).

**Table 3:** Comparison of mean of metals for 4 ecotypes *C. procera, E. alba, P. nodiflora* and *R. sceleratus* with respect to different sites.

Site	Cd (ppm)	Cr (ppm)	Cu (ppm)	Fe (ppm)
Non-polluted	0.062 ± 0.004d	0.062 ± 0.037a	0.420 ± 0.099bcd	1.36 ± 0.130de
Marah drain	0.186 ± 0.043ab	0.089 ± 0.017a	0.835 ± 0.045a	2.45 ± 0.308a
River site 2	0.076 ± 0.016cd	0.078 ± 0.012a	0.560 ± 0.068bc	1.49 ± 0.194cde
River site 3	0.079 ± 0.028cd	0.079 ± 0.025a	0.375 ± 0.066d	1.43 ± 0.261cde
Paharang drain	0.163 ± 0.051ab	0.058 ± 0.020a	0.770 ± 0.109a	1.71 ± 0.272cd
River site 4	0.126 ± 0.040bc	0.062 ± 0.030a	0.580 ± 0.103b	1.19 ± 0.244ef
River site 5	0.078 ± 0.030cd	0.117 ± 0.060a	0.480 ± 0.104bcd	0.87 ± 0.147f
Ah.wala drain	0.223 ± 0.036a	0.094 ± 0.021a	0.889 ± 0.097a	2.07 ± 0.287b
River site 6	0.075 ± 0.013cd	0.042 ± 0.007a	0.384 ± 0.050cd	1.72 ± 0.289bc
River site 7	0.061 ± 0.015d	0.038 ± 0.007a	0.322 ± 0.040d	1.36 ± 0.230e
**Site**	**Mn (ppm)**	**Ni (ppm)**	**Pb (ppm)**	**Zn (ppm)**
Non-polluted	0.189 ± 0.058bc	0.494 ± 0.067bc	0.333 ± 0.051c	1.15 ± 0.119cd
Marah drain	0.164 ± 0.024bc	0.655 ± 0.077a	0.727 ± 0.096a	1.41 ± 0.136b
River site 2	0.151 ± 0.044c	0.415 ± 0.058bcd	0.466 ± 0.086bc	1.05 ± 0.094de
River site 3	0.137 ± 0.035c	0.376 ± 0.065cde	0.414 ± 0.097bc	1.10 ± 0.129d
Paharang drain	0.252 ± 0.053ab	0.535 ± 0.087ab	0.545 ± 0.123b	1.40 ± 0.121bc
River site 4	0.180 ± 0.028bc	0.413 ± 0.090bcd	0.412 ± 0.111bc	0.84 ± 0.116ef
River site 5	0.112 ± 0.024c	0.315 ± 0.069de	0.364 ± 0.090c	0.67 ± 0.130f
Ah.wala drain	0.304 ± 0.055a	0.652 ± 0.067a	0.717 ± 0.103a	1.90 ± 0.133a
River site 6	0.179 ± 0.049bc	0.279 ± 0.046de	0.374 ± 0.065c	1.15 ± 0.063d
River site 7	0.108 ± 0.025c	0.248 ± 0.038e	0.347 ± 0.068c	0.93 ± 0.083de

**Notes.**

Means sharing similar letter in a column are statistically non-significant (*P* > 0.05).

Threatening metal concentrations in plants were checked in four ecotypes in four seasons and it was noted that the highest content of cadmium was present in *P. nodiflora* at 0.161 ppm. The highest chromium concentration was also accumulated in *P. nodiflora* at 0.104 ppm. The concentration of copper was as high as 0.665 ppm in *C. procera*. Moreover, an elevated iron rate was measured in *E. alba* at 2.11 ppm. The maximum Mn contamination level was calculated in *C. procera* at 0.264 ppm. Nickel excess concentration was present in the components of *R. sceleratus* with value of 0.542 ppm. Similarly, the maximum lead concentration was determined in *E. alba* at 0.695 ppm in four seasons. Among the four ecotypes under study, the zinc concentration was observed at its maximum in *P. nodiflora* at 1.38 ppm and the lowest concentration was reported in *C. procera* at 0.81 ppm. Seasonal variations in heavy metal concentrations among the four ecotypes being studied were also significant (Table 4).

**Table 4:** Comparison of mean of different metals for 4 ecotypes, *C. procera, E. alba, P. nodiflora* and *R. sceleratus* with respect to plant components.

Plant	Cd (ppm)	Cr (ppm)	Cu (ppm)	Fe (ppm)
*C. procera*	0.034 ± 0.007b	0.041 ± 0.008b	0.665 ± 0.060a	0.45 ± 0.053c
*E. alba*	0.105 ± 0.012a	0.062 ± 0.016ab	0.448 ± 0.049b	2.11 ± 0.165a
*P. nodiflora*	0.161 ± 0.030a	0.104 ± 0.020a	0.507 ± 0.059b	1.97 ± 0.138a
*R. sceleratus*	0.152 ± 0.021	0.081 ± 0.023ab	0.624 ± 0.063a	1.74 ± 0.127b

Plant	Mn (ppm)	Ni (ppm)	Pb (ppm)	Zn (ppm)
*C. procera*	0.264 ± 0.034a	0.382 ± 0.052b	0.117 ± 0.017c	0.81 ± 0.073c
*E. alba*	0.189 ± 0.017b	0.370 ± 0.037b	0.695 ± 0.056a	1.21 ± 0.063b
*P. nodiflora*	0.160 ± 0.032b	0.458 ± 0.050ab	0.659 ± 0.064a	1.38 ± 0.111a
*R. sceleratus*	0.098 ± 0.011c	0.542 ± 0.044a	0.410 ± 0.044b	1.24 ± 0.071ab

**Notes.**

Means sharing similar letter in a column are statistically non-significant (*P* > 0.05).

**Figure 6:**
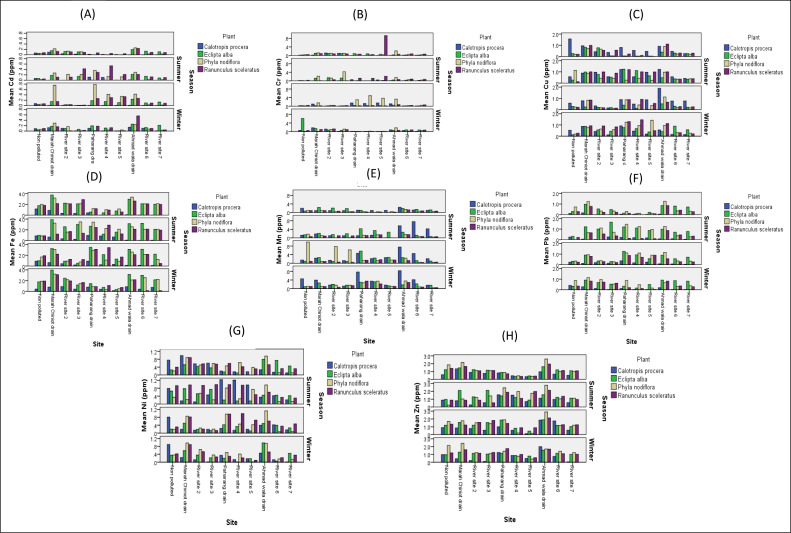
Heavy metals in four ecotypes. Heavy metals Cd (A), Cr (B), Cu (C), Fe (D), Mn (E), Pb (F), Ni (G), and Zn (H) comparison in four ecotypes *C. procera*, *E. alba*, *P. nodiflora* and *R. sceleratus* with respect to seasons at non-polluted, three drains and six river sites.

In seasonal variations in metals at non-polluted, drains and six river sites, results suggest that the *P. nodiflora* has the highest concentration of Cd in the autumn at drains and the lowest in the summer at a non-polluted site (Fig. 6A). The graph for Cr indicates that ecotype *R. sceleratus* concentration was highest in spring at River Site 5 (Fig. 6B). In a comparison of metals in four ecotypes being studied, the high concentration of Cu in *C. procera* was found in spring at non-polluted site, followed by drain sites. The concentration of Cu in *E. alba* with respect to seasons was high in the summer at drains (Fig. 6C). The Paharang drain was most contaminated with respect to Fe content in four ecotypes (Fig. 6D), while Mn highest contents were found in autumn season in *P. nodiflora* (Fig. 6E). In summer season the Pb highest accumulation was calculated at polluted sites as compared to non-polluted site (Fig. 6F). Most of the selected plants showed high value of Ni in spring and summer (Fig. 6G), while highest concentration of Zn was documented in Ahmad wala drain in four ecotypes (Fig. 6H).

### Metal transfer factor (MTF) for four plants under study

The metal transfer factor (MTF) describes the bioavailability of heavy metals to plants. In the calculations for the soil-to-plant metal transfer factor (MTF) as the ratio of plants to metal concentration in soil, the following equation was used (Cui et al., 2004).

\begin{eqnarray*}\text{MTF}=\text{C plant}/\text{C soil}. \end{eqnarray*}

The movement of metals from soil to plants and then to animals is influenced by a number of factors, one of which is the seasonal factor (Osobamiro & Adewuyi, 2015). The difference in bio-concentration factors might be due to the physicochemical parameters of the soil, the binding of metals to plant roots, and the type of plant species (Jackson, 1973). The transfer factor for four plants being studied at ten different sites was determined. The results suggest that TF was significant at polluted sites and TF for Cd and Cr was measured high at River Site 7. The biotransfer factor of heavy metals was calculated highest at River Site 6 for Cu. Fe was significantly higher at River Site 2 and indicated Fe accumulation in plant components.

The metal transfer factor for *C. procera, E. alba, P. nodiflora* and *R. sceleratus* were noted for all ten sites, and the bioconcentration factor for Cd in this finding was greater than 1 in selected plants, which indicates efficient transfer of metals from soil to plants. The bioconcentration factor for chromium in the present finding was also greater than 1 in selected plants, which indicates efficient transfer of chromium from soil to plants. The value of the MT factor greater than unity indicates that plants can accumulate heavy metals, while the MTF less than unity showed that plants just absorb metal (Singh et al., 2010).

The metal transfer factor in *E. alba* showed the highest value of Fe at River Site 2 at 1.788. Mn was present in amounts greater than 1 only in *C. procera* at River Sites 3, 5 and 7. The concentration of Mn in the other three ecotypes was recorded lower than 1 at all sites. This study showed that the Ni and Pb values for the three ecotypes were lower than 1 at all sites, and only *R. sceleratus* was exceptional. The highest Ni value was calculated in *R. sceleratus* at 1.18 at River Site 7. In four ecotypes the Pb values at all sites was calculated lower than 1. MT for Zinc at River Site 3 was highest in four ecotypes under study.

### Comparison of metals in 4 studied ecotypes at non-polluted, drain and river sites

**Figure 7:**
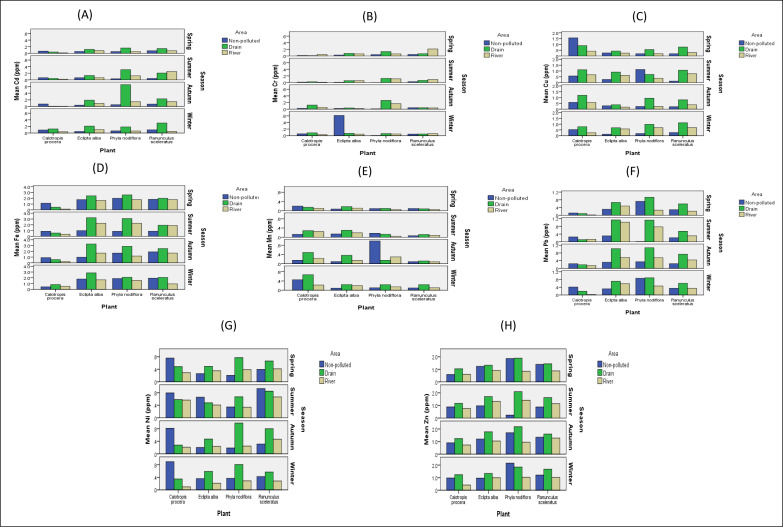
Heavy metals comparison at non-polluted, drains and river sites. Heavy metals Cd (A), Cr (B), Cu (C), Fe (D), Mn (E), Pb (F), Ni (G), and Zn (H), comparison in four ecotypes, *C. procera*, *E. alba*, *P. nodiflora* and *R. sceleratus* with respect to seasons at non-polluted, three drains and six river sites.

The highest concentration of Cd was present in the autumn at three drains in ecotype *P. nodiflora* (Fig. 7A), while the highest accumulation of Cr was noted in *E. alba* in non-polluted site in winter season (Fig. 7B). Overall, Cu concentration was high in drains throughout the year (Fig. 7C). The highest concentrations of Fe in *E. alba* was associated with seasons at drains (Fig. 7D).

The Mn concentration in *E. alba* was high at non-polluted site in the autumn, while it was decreased at a non-polluted site in the spring. Moreover, *P. nodiflora’s* maximum contamination level was present in autumn at non-polluted site (Fig. 7E).

The high concentration of Pb in *C. procera* was reported at non-polluted site in the winter and autumn. In *E. alba,* at drain sites, the highest concentration of Pb was reported. The overall highest concentration of Pb was reported in *P. nodiflora* at drain sites in four seasons. The *R. sceleratus* had the highest Pb concentration at drains in all seasons. Data showed that the highest concentration of Pb was observed in *E. alba, P. nodiflora*, and *R. sceleratus* at drains and was decreased at six different river sites (Fig. 7F). Among comparisons of non-polluted, three drains and six river sites, the value of Ni in *C. procera* was reported high at a non-polluted site in four seasons, while the maximum value of *E. alba* was also reported at a non-polluted site in summer. Overall, the highest concentration of Ni was present at the drain sites. In *P. nodiflora* and *R. sceleratus* the highest concentration of Ni was also present at drains (Fig. 7G). In *C. procera, E. alba,* and *R. sceleratus,* the highest concentration of Zn was present at drains in four seasons while in *P. nodiflora* in non-polluted site the Zn concentration was high in winter (Fig. 7H).

### CCA of physiological and biochemical attributes

The canonical correspondence analysis (CCA) was performed separately for each season and for each site to track the change in physiological and biochemical parameters and heavy metals on a spatial and temporal scale. Some physicochemical parameters such as TDS, Na and SAR were in high correlation with Site 9, which was plotted with Mn, Cu, Cd, Ni, Zn, and Fe. Similarly, most of the metals were highly associated with River Site 6 and Ahmad wala drain.

The triplot for the month of June was much more complex than triplots for the month of March. In summer, the correlation was highly present at River Sites 4, 5 and 6 in the physiochemical parameters of water and soil, which were clustered at the centre, while metals such as cadmium and chromium were found in correlation with the Paharang drain, while metals like nickel and zinc were plotted in correlation with Site 6 and Site 9.

**Figure 8:**
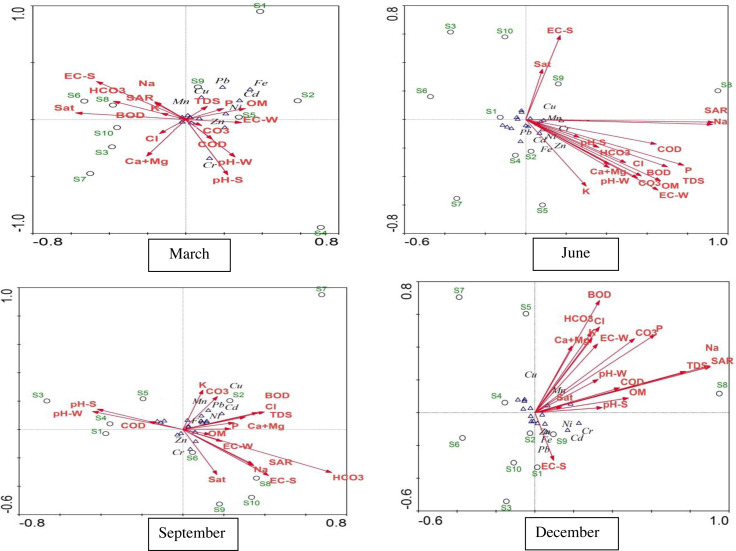
CCA triplots for physiological and biochemical attributes of different study seasons on temporal scale. CCA triplots for physiological and biochemical attributes of different study seasons on temporal scale. March (spring), June (summer), September (autumn), and December (winter). S1 (River Site 1, non-polluted), S2 (Marah Chiniot drain), S3 (River Site 2), S4 (River Site 3), S5 (Paharang drain), S6 (River Site 4), S7 (River Site 5), S8 (Ahmad wala drain), S9 (River Site 6), S10 (River Site 7).

Autumn showed much more variation compared to other seasons. Water and soil physicochemical parameters were present in association with Site 5 and Site 2. Similarly, Site 6 was plotted towards Zn and Cr mainly and showed some association with saturation of soil. Most of the metals were found in association with Sites 8 and 9. Heavy metals were highly correlated with Site 9 and showed some association with Site 2. Most of the physiochemical parameters for water and soil were plotted towards Site 8 and Site 2 (Fig. 8).

## Discussion

### Water and soil study

It was hypothesized that the three drains coming from Faisalabad industries carry polluted water. To test this hypothesis, soil and water samples were collected from the drains and river Chenab and subjected to different tests. High levels of pollution were recorded in both soil and water samples collected from the Chenab River and its associated drains. One site was selected as a non-polluted site before the entry of the first drain into the Chenab River.

Among the attributes of the water being studied, pH did not vary significantly across the non-polluted site, drains and river sites. However, at drains pH of the water was higher, which can be attributed to the presence of pollutants in the drainage water from the Faisalabad industries, which are considered a major contributor to pollution. Drains along river sites are facing severe degradation in water quality because of industrial effluents. The water quality was relatively better at a non-polluted site 20 km above the first drain. Water quality was degraded from the Marah Chiniot drain point source to the Ahamd wala drain and its associated river sites (Sites 6-7) because of pollutants from drains. The electrical conductivity (EC) of water indicated a higher contamination level and pollution stress at all sites except River Site 1, a non-polluted site and Site 7, where river water flow was high. Such results were also reported earlier by Farid et al. (2013). The EC of water samples from all drains exceeded the limit set by FAO for irrigation water.

Spatial variations in the water quality of drains and associated river sites were due to various parameters such as total dissolved solids (TDS), chemical oxygen demand (COD), biochemical oxygen demand (BOD), electrical conductivity (EC), and heavy metals like cadmium (Cd), chromium (Cr), copper (Cu), iron (Fe), manganese (Mn), nickel (Ni), potassium (K), phosphorous (P), sodium (Na), and zinc (Zn). It was reported that concentrations of all these metal ions in the drain effluents were above the recommended NEQS limits. The problem of pollution in the Faisalabad industrial polluted area has been reported previously by (Imtiazuddin, Mumtaz & Mallick, 2012). However, the problem with respect to seasons was noted to be more severe at drain sites.

The contribution of parent rock material that was loaded with Fe, was evident in the concentration of Fe in the studied samples at River Site 1 Chenab Nagar, as Chenab Nagar is a mountainous area. A high level of pollution was confirmed at three drains and associated river sites. Such a high level of pollution in drains and rivers can be attributed to the locality of the area. This fact is also supported by the high EC of samples from drains and some river sites. The problem of industrial pollution from industrial areas has been reported previously by Akey & Appel (2021), and Farid et al. (2013). However, due to drainage into the Chenab River, the problem is more severe. In addition to the effects of EC, metal toxicity was noted in most of the drains and river sites. Among the heavy metals, iron was noted at the highest levels at drains and River Site 1, which is in the mountainous area. The COD and BOD of the water in three drains were also unsuitable for the health of the flora of the area and the vegetation in drainage basins. The vegetation, which was in direct contact with effluents, showed stunted growth. Furthermore, at three drains and most river sites, most metals, such as Cd, Cu, Mn, Ni, Pb and Iron were present in toxic concentrations that exceeded FAO limits. The presence of these metals in industrial pollution has also been reported by Sharma et al. (2021) and Imtiazuddin, Mumtaz & Mallick (2012).

The analysis of soil included a non-polluted site, the pH of the soil did not vary much; however, the EC of the soil was high at the non-polluted site. On the other hand, saturation of the soil did not vary significantly across the selected sites. It was observed that the availability of organic matter was twice as high at drains compared to river sites and the concentration of phosphorus was also much higher at drains compared to river sites. The potassium value was also higher at drains and their associated sites compared to a non-polluted site. Higher content of organic matter and phosphorus at drains indicates the flourished growth of plants along the drains and associated river sites. The potassium concentration was also high at drains. The higher amount of these minerals indicates the higher vegetation density at these sites, which was noted in the second phase of the study. Among the heavy metals, the amount of iron was noted to be high at all sites along River Site 1, which showed the peak values of iron along the drains. At all sites studied, the amount of iron was the highest, followed by lead. The concentration of zinc was also higher at all sites, and its value was greater than 1 at all 10 sites. Their source has been described earlier, the drainage water from industrial pollution. The heavy metals come with industrial pollution drainage and can be deposited in soil or settle in the beds of drains. Such pollution has been reported by Raza et al. (2021) and Akhtar et al. (2005). On the other hand, the large affected areas show the seriousness of industrial pollution and its potential to distort the vegetation structure on a large scale. The threatening effects of industrial pollution on vegetation have been described by many studies, such as Cordova (2021), Sorrentino et al. (2018) and Lin et al. (2007), as industrial pollution removes or severely damages vegetation through bioaccumulation. Therefore, it is crucial to implement effective measures to reduce industrial pollution and protect our environment.

Keeping all the attributes under study in water and soil in view, and with the help of CCA and ANOVA both in soil and water, the major cause of pollution was found to be a problem of metals, followed by the spread of industrial pollution to large areas with the help of drainage from industrial areas and metal toxicity mainly due to lead, iron and zinc. The current study also suggests that drains were found to be a major contributor to the pollution at all sites except River Site 1.

### Study of heavy metals along soil to plant MTF in selected plants

The study of soil and water in phase 1 revealed that among the commonly occurring heavy metals in industrial effluents, only lead, manganese, iron, nickel and cadmium were found in toxic amounts. Among these five metals, lead and cadmium, along with iron toxicity, were found at all sites, but lead and cadmium were not in toxic concentration at River Site 1, which was free from industrial pollution. However, the plants were tested for all eight metals to have any indication of hyperaccumulator species among the selected plants. For this purpose, the soil-to-plant metal transfer factor (MTF) was also calculated and was subjected to CCA to see if there was any association between the metals in the soil and plants.

Metal transfer factor for *C. procera, E. alba, P. nodiflora and R. sceleratus* were noted for all ten sites and the results suggest that the Cd concentration transfer factor in *C. procera* was present as high as 2.83 at River Site 3, followed by River Site 2 with value of 2.26, and the decreased level was noted at Site 2 with value of 0.09. In *E. alba,* the highest 8.75 MT was measured at River Site 3, followed by Site 3 at 7.00. Similarly, at Site 8, 1.32 TF was calculated, and the decreased concentration was noted at Site 8. The Cd contents in *P. nodiflora* were calculated to be greater than 1 at river sites 4, 6 and 7. In *R. sceleratus*, the cadmium concentration was as high as 12.00 at River Site 3, followed by Site 3 and the lowest at Site 8. The overall cadmium contents measured for TF were the highest at River Site 3 in all plants.

The MT value for chromium was recorded highest in *C. procera* at Site 1 (10.31), while recorded lowest in *P. nodiflora* at Site 8. Overall, in all four plants (*C. procera, E. alba, P. nodiflora* and *R. sceleratus), the* value of chromium was higher than 1 and showed that chromium was accumulated in all these plants at all sites. The Cu measured in *C. procera* at Site 1 and Site 6 showed values greater than 1, while *P. nodiflora* and *R. sceleratus* at Site 1. Therefore, the value was higher at Site 1 and the lowest measured at Site 8 in *E. alba.* The bio-concentration factor for cadmium and chromium in the present findings was greater than 1 in selected plants, which indicates efficient transfer of metals from soil to plants Wang et al. (2015).

The metal transfer factor for the plants being studied was measured at all ten sites. A ratio of Fe to the metal transfer factor in *C. procera* was found to be highest at Site 3 (0381) and lowest at Site 9. The MT measured for *E. alba* showed the highest value of Fe at Site 3 (1.788) and the lowest at Site 1. Similarly, *P. nodiflora* showed a peak of Fe at Site 3 (1.669) and the least value at Site 9 (0.375). The ecotype *R. sceleratus* showed the highest value at Site 3 (1.47) and the lowest at Site 8 (0.332). MTF measured for Mn was greater than 1 only in *C. procera* at Sites 4, 7 and 9, while it was lowest in *C. procera* at Site 10 as 0.075. Among the other three ecotypes, Mn was recorded below 1 at all study sites. The overall bio-concentration factor for Fe at some sites was greater than 1, indicating its greater availability to plants and lower retention in the soil. Ahmad et al. (2018a) also recorded higher transfers of Fe (6.26−6.79) from soil to plants.

The value of an MT factor greater than unity indicates that plants can accumulate heavy metals, while an MTF less than unity shows that plants just absorb metal (Singh et al., 2010). Calculations for MT show that the Ni and Pb values for the three ecotypes were lower than 1 at all sites except *R. sceleratus.* The nickel’s highest value was calculated in *R. sceleratus* as 1.18 at Site 10 and the lowest in *C. procera* as 0.27 at Site 5. The TF for Pb calculated at all sites in all samples was measured lower than 1. Zn’s highest value for MT was reported at Site 4, as 1.03, followed by Site 10 as 1.01 in *P. nodiflora,* and the decreased in *C. procera* at Site 5. The results suggest that most of the plants under study were hyperaccumulators, as the value of MTF was higher than 1 in all plants at different sites for different metals.

## Conclusion and Recommendation

At the end of the study, it has been concluded that a significant level of pollution is present at the study sites of the River Chenab and the three associated drains carrying effluents from the industrial areas of Faisalabad, Punjab, Pakistan. The pollution is present in the form of high metal toxicity due to the presence of a large number of effluents along drains. Metal toxicity, especially the lead, iron, zinc, chromium, and cadmium toxicity, possibly due to effluents from the industries, was higher. The water and soil quality was degraded as the quantity of TSS, TDS, and SAR increased because of the large quantity of effluents from the Faisalabad industries flowing into an enormous area of drains and the river Chenab. Moreover, the study has revealed that industrial pollution, which is a major contributor to pollution, is severely damaging the native vegetation of the area. Despite higher levels of pollution, four plants (*i.e., Calotropis procera, Phyla nodiflora, Eclipta alba* and *Ranunculus sceleratus)* were found growing at all sites. However, phytoremediation by all studied plants has been identified as the most dominant, acclimatized species of the area that can be used for the revegetation and reclamation of industrial pollution.

##  Supplemental Information

10.7717/peerj.15565/supp-1 Supplemental Information 1 Water analysis metal raw data

10.7717/peerj.15565/supp-2 Supplemental Information 2 Plants metal analysis

10.7717/peerj.15565/supp-3 Supplemental Information 3 Soil metal analysis

10.7717/peerj.15565/supp-4 Supplemental Information 4 Water analysis Water physicochemical parameters

10.7717/peerj.15565/supp-5 Supplemental Information 5 Soil parameters Soil physicochemical parameters

10.7717/peerj.15565/supp-6 Supplemental Information 6 Soil and water combined soil and water combined

## Additional Information and Declarations

### Data Availability

The following information was supplied regarding data availability: The raw data are available in the Supplemental Files.
